# Extracting Forest Parameters based on Stand Automatic Segmentation Algorithm

**DOI:** 10.1038/s41598-020-58494-6

**Published:** 2020-01-31

**Authors:** Pengxiang Zhao, Linghan Gao, Ting Gao

**Affiliations:** College of forestry, Northwest A&F University, Yangling, 712100 China

**Keywords:** Environmental impact, Environmental impact

## Abstract

Forest stand segmentation is a critical process for forest management and inventory. The forest stand segmentation accuracy will determine the forest stand level parameters quality. In this study, we developed an automatic forest stand segmentation algorithm based on ArboLiDAR, a software used to process Light Detection and Ranging (LiDAR) point cloud data. We then optimized the parameters for the algorithm to the Dayekou forest area on Qilian Mountain in China to find the most suitable parameters for automatic stand segmentation. Further, we extracting the forest parameters at the stand level based on Bysh method. Our results showed that the limited region growing method based on the gradient is the most suitable one for analyzing automatic stand segmentation in the studied area. Among our tested parameters groups, the fifth group contains the optimal parameters for the studied area. In addition, for forest parameters, the R^2^ of mean height (H), average diameter at breast height (D), basal area (G), and Stand volume (V) is 0.744, 0.720, 0.562, 0.696, respectively. The RMSE value is 5.24%, 28.57%, 19.93%, and 17.66%, respectively. Our study serves as a technical basis and reference for future studies that perform more efficient analyses on forest resource inventory in China.

## Introduction

Forests play a vital role in their areas with economically important products such as timber and several environmental benefits such as air quality improvement, water-flow filtering, and climate regulation^1^. Reliable and up-to-date information on forest structure helps the management of forest resources more effectively^2^. Scanning Airborne LiDAR is an active remote sensing technique not only able to capture the entire forest canopies three-dimensional structure with high precision but also proves to be more stable than conventional aerial or satellite spectral imaging^3–5^. This makes Airborne LiDAR a promising system for investigating variations in forest structure^6^.

Applying Airborne LiDAR to retrieve forest parameters can be dated back to the early 1980s^7,8^. Many studies identified significant relationships between Airborne LiDAR and forest parameters field measurements such as height, basal area. It can also support the generation of vegetation structure and biomass broad-scale assessments^9–13^. In Garcia’s paper^14^, the different biomass fractions of a Mediterranean forest (the total above ground, the branches, and the foliage) was estimated using LiDAR height, intensity data and a-priori information on specific species, achieving R² values greater than 0.85, 0.80, and 0.90 for black pine, Spanish juniper and Holm oak, respectively. Silva *et al*.^15^ developed multiple regression models predicting Eucalyptus plantations stem volume from selected LiDAR metrics, with a model coefficient overall determination of 0.87, and a root mean squared error of 27.60m^3^ha-1.

Traditional regression methods used in retrieving forest parameters from LiDAR data often require a significant amount of plot data^16^. However, obtaining ground truth data necessary can be expensive. One particular regression method, known as Sparse Bayesian regression, has proved to be more effective than other regression methods with only a limited number of ground truth samples need to be acquired for analysis^17^. Nevertheless, to date, there are few methods that have been closely examined for how it might contribute to forest parameter estimation.

In this paper, we investigated the Sparse Bayesian regression model that was first developed by Tipping^18,19^ and assessed how effectively it might be applied to Airborne LiDAR data in order to generate accurate forest structure parameters. This paper also explored how this proposed method might be integrated successfully with the outputs of commercial software such as ArboLiDAR.

Forest stand segmentation is a critical task for stand-level forest parameters extraction because the segmentation process results are used as a fundamental input for the additional process required to extract some parameters. Both forests stands segmentation and parameters extraction are examined in this paper. Forest stands are basic units and can be defined in terms of tree species or tree maturity. From a remote sensing point of view, forest stand segmentation should be carried out according to a specific segmentation algorithm. Many studies have focused on segmentation algorithms development based on remote sensing data. Several automatic image segmentation approaches were developed, such as (i) thresholding technique, (ii) boundary-based method (edge-detection), (iii) global optimization approach based on energy functions or Bayesian^20,21^, (iv) region-based method^22^, (v) watershed algorithm^23,24^, and (vi) hybrid technique^25,26^.

Although there are many algorithms for segmentation, most of them are aimed at images, and few studies have been conducted on specific regions, especially forest areas. With LiDAR technology application, research on segmentation algorithms based on LiDAR data and multi-source remote sensing data has attracted a lot of attention.

Sullivan *et al*.^27^ proposed the low-density airborne LiDAR use for object-oriented image segmentation and supervised classification. The segmentation is performed using a region growing approach. Spatially adjacent pixels are grouped into homogeneous discrete image objects or regions. Hay *et al*.^28^ developed Multiscale Object-specific Segmentation (MOSS) and showed that it can be used to automatically delineate objects ranging from individual tree crowns to forest stands.

Pyysalo *et al*.^29^ carried out a single tree crowns reconstruction from laser scanner data using the obtained vector model for feature extraction. The results showed that dense laser scanner data can be used to extract the details of upper forest canopies and tree height information. The lower crown found less detail and the parameters extracted from that part were less accurate, but trendsetting. Morsdorf *et al*.^30^ introduced a new approach to derive the structure of the upper canopy by segmenting single trees from small-footprint LiDAR data and deducing their geometric properties. The objective is to reconstruct a geometric forest scene using a paraboloid model and information about tree position and height, crown diameter and base height.

Zhang *et al*.^31^ used an object-based algorithm to classify tree species using LiDAR and hyperspectral data for two study areas. Their results show that individual tree species can be identified in urban forests using the object-based algorithm with multi-source remote sensing data fusion. Dalponte *et al*.^32^ defined a novel adaptive thresholding method that can remove any subjectivity in the thresholding process by using hyperspectral and airborne LiDAR for Airborne Remote Sensing System (ALS) data in the automatic individual tree crown (ITC) delineation.

In addition to the above studies, there are many LiDAR applications in forestry inventory^33–36^. However, even though this method was successfully applied to segment forest stands, it remains an open question as to whether it can generate a similar success when merging Airborne LiDAR data with aerial photos, where there are usually only three color bands R(red), G(green), and B(blue) available. This study investigated the performance of “gradient and region growing algorithm” (GARGA) method in stands segmentation with LiDAR data and aerial image data based on ArboLiDAR (Arbonaut).

In summary, the objectives of this paper are:1) evaluate whether Sparse Bayesian regression is effective in estimating forest stand parameters. 2) assess region growing algorithm applicability for stand segmentation when combining Airborne LiDAR data with aerial photos. 3) evaluate ArboLiDAR applicability to conduct forest inventory in the north-west of China.

## Materials and Methods

### Study area

The study area is located in the Dayekou forest area on Qilian Mountain in Zhangye city, Gansu Province. The center coordinates of the study area are approximately 100°15′ E, 38°32′ N. The study area contains the water conservation forest in the Heihe basin, which belongs to the Qilian Mountains National Nature Reserve, and also contains the Heihe integrated remote sensing forest hydrological experiment station. The LiDAR aerial data was acquired on June 23, 2008. The Heihe integrated remote sensing experimental data and the Dayekou basin flying airborne LiDAR data sets were derived from Yong *et al*.^37^.

**Figure 1:**
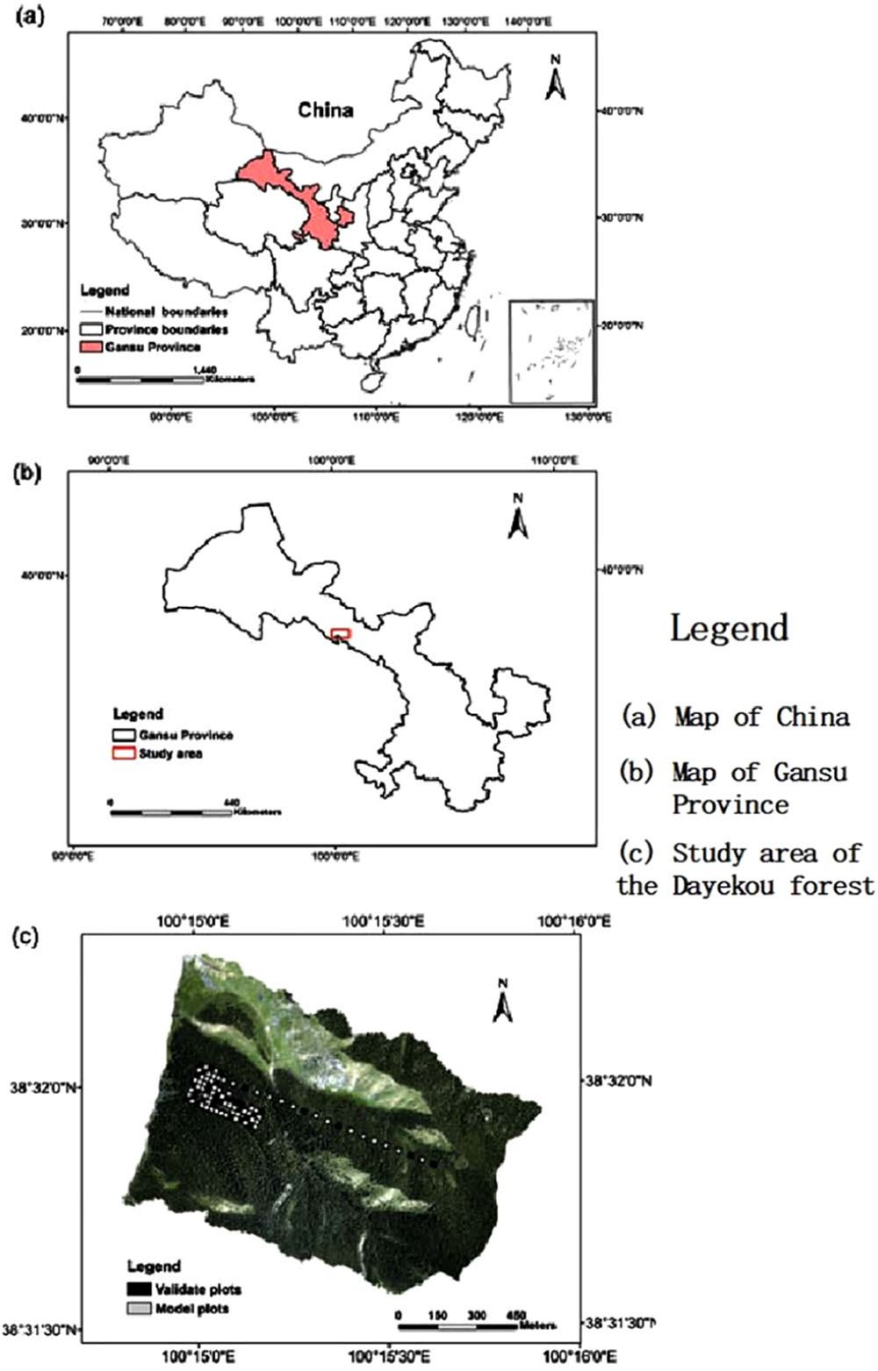
Map of the Dayekou forest in Zhangye city, Gansu Province, northwest China, showing the study area location.

In this study, the field plot data acquisition occurred between June 2008 and August 2010. Since the trees growth rate is slow within two years, it can be used as verification data for our study. In the forest area, plot sampling was conducted along a 1000-meter transect and a supersampling area of 300 m × 340 m. Along the 1000-meter transect, 20 m × 20 m square plots were established at an interval of 50 m, and 20 plots were established in total. Among the established plots, 19 plots were selected for our study. The super sample was divided into 255 20 m × 20 m plots, and 75 plots were selected to study. Therefore, a total of 94 plots were selected for the study, 60 of which were used to build the model, and 34 of which were used to test the model (see Fig. 1).

The satellite imagery is obtained by CCD camera on unmanned aerial vehicle(UAV). The satellite imagery and lidar data were obtained by the UAV flight of China Academy of forestry in 2008. The website is http://westdc.westgis.ac.cn/search?q = LiDAR + CCD.

### Method

#### Field data

The central location of each plot was recorded using a Differential Global Positioning System (DGPS) devices and corrected using post-processing techniques. Diameter at Breast Height (DBH), tree height and crown of all individual tree in each plot were rerecorded. Forest stand characteristics: Lorey’s mean height (H), average diameter at breast height (D), basal area (G), and Stand volume (V) were calculated using previously established models based on the measured tree-level information^38^ (Eqs. 1–4), respectively.

1 $$H=\frac{{\sum }_{i=1}^{N}{h}_{i}{g}_{i}}{{\sum }_{i=1}^{N}{g}_{i}}$$

2 $$D=\sqrt{\frac{4\bar{g}}{\pi }}=\sqrt{\frac{4}{\pi N}G}=\sqrt{\frac{4{\sum }_{i=1}^{N}{g}_{i}}{\pi N}}=\sqrt{\frac{{\sum }_{i=1}^{N}{{d}_{i}}^{2}}{N}}$$

3 $$G={\sum }_{i=1}^{N}{g}_{i}={\sum }_{i=1}^{N}(\pi \times {(\frac{{d}_{i}}{2})}^{2})$$

4 $${\rm{V}}=\frac{({\sum }_{{\rm{i}}=1}^{{\rm{N}}}(0.000053108582\times {{d}_{i}}^{1.778667}\times {{{\rm{h}}}_{i}}^{1.1280516}))}{400}\times 10000\,$$

where hi, ${g}_{i}$ and ${d}_{i}$ represent the height, the basal area and the diameter of i-tree in each plot, respectively. N represents the number of stems in a plot. For this study, $\bar{g}$ denotes the mean of the basal area in one plot. The unit parameter of H, D, G, V is m, cm, m^2^, m^3^/ha, respectively.

#### Data preparation for stand segmentation

Forest stands are the basic units used in forest management. They are used as inventory units and operational units. The automatic segmentation approach developed in this study took into account the timber size and forest density. This information was derived from LiDAR data. Other information can be obtained from aerial images.

In general, segmentation was done using raster data as input. Therefore, we created suitable raster data using LiDAR information. The raster pixel size can be set to 4 m × 4 m, which can show the high quality of different vegetation parameters. Smaller pixel sizes would provide too detailed information about the forest structure small-scale variation. We were interested in generalized forest stand level information instead of information about a single tree and a small gap in the forest. In this study, we used raster data for the stand height, and we derived the stand density from LiDAR data and aerial image raster classification.

The vegetation height can be derived directly from LiDAR data. It is often more efficient to create a raster with height attributed to each pixel, but in this study, a percentile height (usually 85 percentile) was used instead of the maximum height to eliminate outliers. Vegetation height at the 85 percentile means the height where 85% of the all LiDAR data hits lie below that height. Each height raster pixel contains the actual height value in the given percentile. The height raster has a good correlation with the average tree height.

The density raster describes the ratio between the number of LiDAR vegetation points and the total number of LiDAR points within a pixel. The vegetation points are those with altitude (Z value) higher than the height at the given percentile. The pixels value range in the density raster is from zero (no forest) to one (very dense forest). The density raster, in particular, has a good correlation with forest basal area.

The aerial images classification describes the information about vegetation species and land-cover. The maximum likelihood method was used to classify the original aerial image. It was assumed that each class in each band is normally distributed, and the likelihood of the given pixels in the training sample can be calculated. Eventually, the pixels were merged to the class of maximum likelihood. The classification band has a good correlation with forest species composition.

After obtaining the raster data, we modified the rasters for better segmentation. In this study, we used median-filtering and mean-shift filter methods to modify the raster data. The median filtering method applies a moving window and calculates a median of the values within that window to the center pixel of the window in the input raster. The purpose of this method is to eliminate isolated noise points by creating a greater difference in pixel value close to the surrounding pixels. The mean-shift filtering method is an iterative filtering method that filters the image in a given spatial and spectral radius. It keeps the nonlinear image borderline. Finally, we generated a composite band of the height raster, density raster, and classification raster for the stand segmentation.

#### Algorithm of stand automatic segmentation

The automatic segmentation method iteratively created the segmentation over many phases. First, a gradient image of the segmentation raster was produced. A gradient showed the variation of the values in the area of interest. When creating the gradient image, the band-wise weight can be given. Meanwhile, seed points were placed in suitable areas during every iteration. The seed points were always the local minimal points within a pixel area.

Second, a limited iterative region growing method was used to find areas similar to the seed point environment in every iteration step. Firstly, this method was used to find the promising seed points and segment out the homogenous areas around those seeds using the region growing algorithm. Secondly, new seeds were added only on the unsegmented areas by gradually decreasing the seed-finding criteria; then, the region growing was re-run, starting from the combined seeds from all previous iterations^39^.

Finally, we merged the insufficiently small pixels of the resulting segmentation. In the merging analysis, the mean and standard deviation of the sum band were calculated. The segments were then merged with the neighboring segments, and the most optimal segment was based on these values. If the most optimal neighbor was below the band-unique merging threshold, it can be combined with the segment. If the initial segment was the most optimal for the neighboring segment, it can be merged as well. After merging, the mean and standard deviation were recalculated.

The automatic segmentation method did not have a universal parameters combination that can be applied in all environments. The parameters values depend always on the area inventoried, the segmentation variable raster type, and the requirements for the segmentation. The segmentation algorithm parameters include the gradient-band weight, region growing band weight, priority function, and the competition threshold value. The merging algorithm parameters include the mean difference, standard deviation difference, band weight, maximum area, and small segment area. In this study, we set three parameters groups to a segment based on the field situation and image data. By comparing the stand automatic segmentation results under different parameter settings and manual segmentation results, the most suitable parameter combination setting was found for the study area based on an automatic segmentation algorithm.

#### Forest parameters extracting

Sparse Bayesian regression model is able to automatically apply different weightings according to different variables relevance within an estimation and it has shown good performance when the plot data amount is limited. In this study, a Sparse Bayesian regression model is formulated automatically by an algorithm that compares different weighted combinations of feature values with all other values to derive the weights optimal distribution and a features optimal set. By automating the model formulation and the sample plot selection, this approach offers increased flexibility when it comes to prediction^17^. Four forest parameters included: H, D, G, and V were used as estimated variables. Meanwhile, 76 predicted variables default defined by ArboLiDAR were derived from the Airborne LiDAR data mainly including: different height percentiles for the first-pulse and last-pulse return; the mean height of the first-pulse return above 5 meters (the high-vegetation return); the standard deviation of the first-pulse return; the ratio between the first-pulse return below 1 meter and all of the first-pulse returns; the ratio between the last-pulse returns below 1 meter and all the last-pulse returns; and several intensity-related features. These Airborne LiDAR variables are based on features that were originally described by together with those referenced in the user manual for ArboLiDAR^40^.

In this study, 60 plots of data were selected randomly to construct the Sparse Bayesian model. The model was trained based on the following principal steps: First of all, constructing a model at plot-level which was the basis of how the estimation model was then defined. Adding plots boundary data to the ArboLiDAR project and completing the attribute tables with the estimated variables, which is calculated from the measured plots. Then, predicted variables from the Airborne LiDAR data were calculated. An inventory model was then generated using the measured variables and predicted variables. This process required a few iterations in order to construct a model that could properly reflect the statistics, the plot location, and the overall inventory process. After the process of conducting model at plot-level, the ArboLiDAR software verifies the model by adopting the Leave-One-Out (LOO) method so it decided whether this model was suitable for the forest parameters estimation at stand-level. This study achieves a better result at this step. Thus, the forest inventory results at stand level were estimated based upon the results of segmentation successfully. In order to match the size of the actual field plots, the minimum cells of the segmentation results were re-sized into 20 ×20 meters first. Then LiDAR variables of all cells were calculated. These variables were used as input variables of ArboLiDAR generated inventory results with LiDAR variables of cells as input variables based on the model built. Finally, the inventory results were aggregated to the stands level so that forest parameters of 381 forest stands were obtained.

#### Accuracy evaluation

34 plots of data were selected randomly to validate the performance of this model. Correlation analysis between the measured data and the estimated data were conducted using the open-source software “R3.2.2”, which provides a statistical computing environment^41^. The RMSE% (in the paper, RMSE is defined as a percentage of the mean value), and the Pearson coefficient squared (R^2^), together with Bias% (in this paper, Bias% also defined as a percentage of the mean value), were selected to validate the model^42–44^ (Eqs. 5 and 6), respectively.

5 $$RMSE=\sqrt{\frac{{\sum }_{{\boldsymbol{i}}=1}^{{\boldsymbol{n}}}{({{\boldsymbol{m}}}_{{\boldsymbol{i}}}-{{\boldsymbol{e}}}_{{\boldsymbol{i}}})}^{2}}{{\boldsymbol{n}}}}$$

6 $$Bias=\frac{1}{n}{\sum }_{i=1}^{n}({m}_{i}-{e}_{i})$$

where ***m***_***i***_ represents the forest parameters from the field data measured value; ***e***_***i***_ denotes the forest parameters estimated value according to the Airborne LiDAR data that was generated by the ArboLiDAR system; n is the validation plots number.

## Results

### Data preprocessing

The original LiDAR data point cloud contained many small noise points and different object categories, which need to be processed by the classification filter to obtain better data results for the segmentation operation.

**Figure 2:**
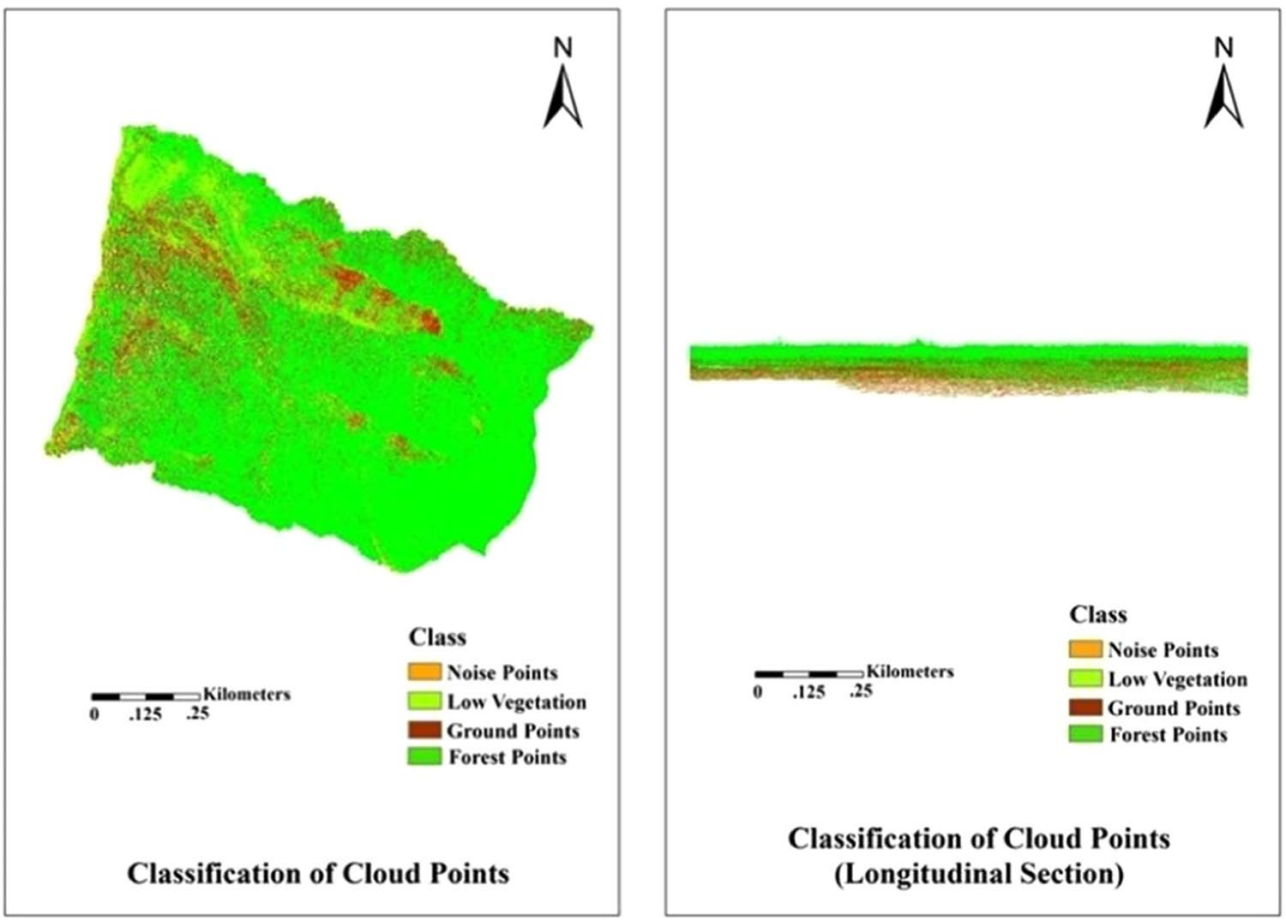
Classification of cloud points (transverse and longitudinal section).

The point cloud data for this area were divided into noise (low and high points), ground points, low vegetation, and forest points based on LiForest (http://greenvalleyintl.com/). They were shown as light brown, dark brown, yellow, and green, respectively, in Fig. 2. In addition, we extracted the digital terrain model (DEM) based on the ground points and processed the elevation normalized operation to eliminate the topography influence for the results of the stand segmentation. We intended to obtain pure height information. It can be seen from the longitudinal section in Fig. 2 that all objects were distributed along the same horizontal line without terrain factors influence, such as slope and elevation.

### Generating composite band (DHH)

In the stand automatic segmentation algorithm, the composite data (height, density, and aerial photo classification, referred to as DHH) were needed. The height raster can extract more accurate height information. The density raster has a strong correlation between stand density, basal area, and volume. The aerial photos classification (1 m resolution) can obtain more accurate object information. The three sets of data synthesis enhanced the overall information and provided a better basis for the automatic segmentation algorithm.

**Figure 3:**
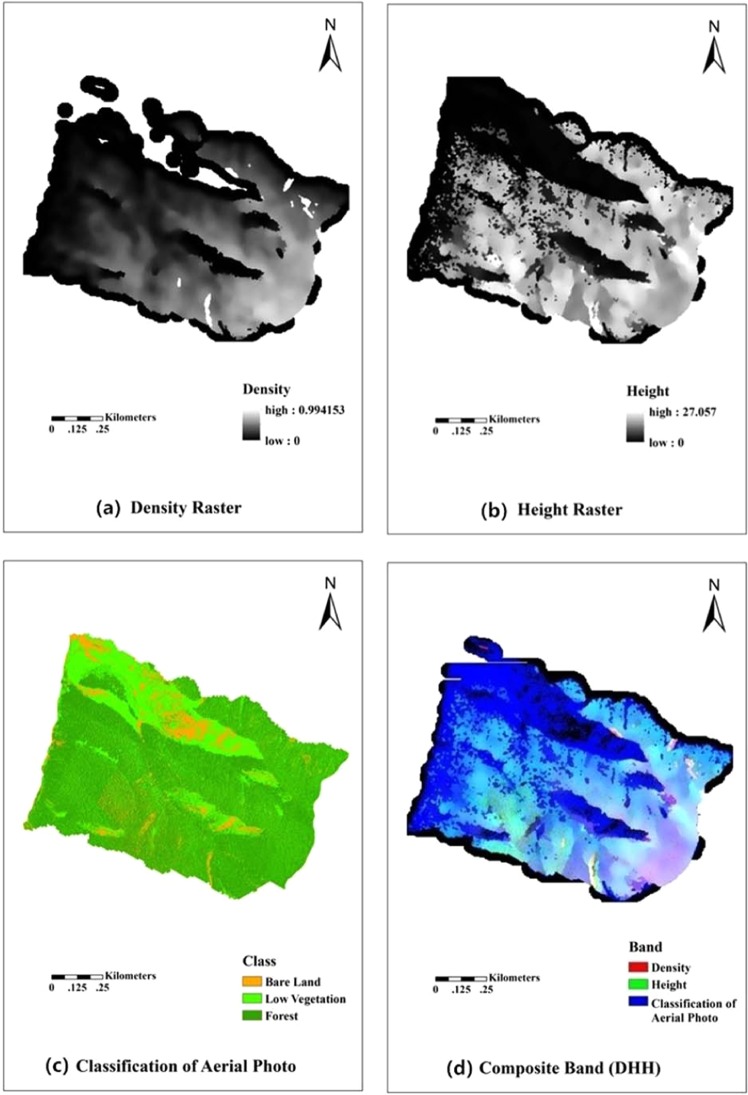
Four maps (**a**–**d**) represent density raster, height raster, classification of aerial photo and composite band (DHH), respectively.

The height and density raster were extracted based on ArcGIS (http://www.esri.com/). The extracted height and density data were filtered using the median filter and mean-shift filter in order to smooth the edges and eliminate the errors in the data. The height range was 0–27.057 m and the density range was 0–0.994153, which are presented in Fig. 3.

The aerial photos classification was achieved based on ENVI (http://www.esri.com/). The objects were divided into bare land, low vegetation (moss and shrub), and forest using the maximum likelihood method, based on the field situation and aerial photo information. In Fig. 3, the classification results were expressed as brown, light green, and dark green. In addition, the classification accuracy was examined using the kappa coefficient. The kappa coefficient was 0.8 for the classification result. Therefore, the classification accuracy was good according to the kappa coefficient evaluation criteria proposed by Cohen^45,46^. In these criteria, the result can be used for correlation analysis and prediction when the kappa coefficient ranges from 0.6–0.8.

The combination of the height, density, and classification data from the aerial images are shown in Fig. 3, composite band (DHH). Then, the stand automatic segmentation operation was accomplished by setting the parameters of segmentation and merging based on DHH.

### Stand automatic segmentation

#### Setting segmentation parameters

The stand automatic segmentation operation included two steps: segmentation and merging. The most critical element of stand automatic segmentation algorithm is the segmentation parameters setting. In this study, we intended to find the optimal segmentation parameters combination using many factors through the multi-parameter settings in order to obtain the best segmentation result for this study area. Stand automatic segmentation can provide a reference for the subsequent development of forest inventory.

Based on the algorithm requirements, the scenario illustrated by the aerial photo, point cloud data, and field investigation, we set up seven sets of segmentation parameters and corresponding merging parameters. This information is listed in Tables 1 and 2.

**Table 1:** Segmentation parameter settings.

		1	2	3	4	5	6	7
Segmentation Parameters	Gradient band weights	0.3/0.3/0.3	0.4/0.3/0.3	0.3/0.4/0.3	0.3/0.3/0.4	0.3/0.3/0.3	0.4/0.3/0.2	0.3/0.3/0.3
	Region growing band weights	0.3/0.3/0.3	0.4/0.3/0.3	0.3/0.4/0.3	0.3/0.3/0.4	0.3/0.3/0.3	0.4/0.3/0.2	0.3/0.3/0.3
	Priority function	0.0/0.1/0.15/0.2/0.3/0.5/1.0				0.0/0.1/0.2/0.3/0.5/0.7/1.0	0.0/0.1/0.15/0.2/0.3/0.5/1.0	
	Competition threshold	0.02						0.04

**Table 2:** Merging parameter settings.

		1	2	3	4	5	6	7
Merging1 Parameters	Mean difference	0.05/1.0/2.0						
	Standard deviation difference	0.0/1.0/0.2						
	Band weights	0.3/0.3/0.3	0.4/0.3/0.3	0.3/0.4/0.3	0.3/0.3/0.4	0.3/0.3/0.3	0.4/0.3/0.2	0.3/0.3/0.3
Merging2 Parameters	Mean difference	0.1/1.5/2.5						
	Standard deviation difference	0.008/1.5/0.4						
	Band weights	0.3/0.3/0.3	0.4/0.3/0.3	0.3/0.4/0.3	0.3/0.3/0.4	0.3/0.3/0.3	0.4/0.3/0.2	0.3/0.3/0.3
Merging3 Parameters	Mean difference	0.15/1.8/3.0						
	Standard deviation difference	0.015/1.8/0.6						
	Band weights	0.3/0.3/0.3	0.4/0.3/0.3	0.3/0.4/0.3	0.3/0.3/0.4	0.3/0.3/0.3	0.4/0.3/0.2	0.3/0.3/0.3
Merging4 Parameters	Mean difference	0.2/2.0/3.2						
	Standard deviation difference	0.02/2.0/0.75						
	Band weights	0.3/0.3/0.3	0.4/0.3/0.3	0.3/0.4/0.3	0.3/0.3/0.4	0.3/0.3/0.3	0.4/0.3/0.2	0.3/0.3/0.3
	Maximum area	50000						
	Small segment area	500						

In Table 1, the three bands weights (density, height, and aerial photo classification) in the gradient algorithm and the region growing algorithm had an impact in the stand automatic segmentation, because different bands had different emphasis and forest information. In addition, the settings of the priority function and competition threshold in the region growing algorithm were needed for the segmentation. During the parameter setting process, we only set up two different sets of priority functions and competition thresholds because the terrain and pixels influence was avoided in early data processing, so those two parameters had little influence on the segmentation results. The key segmentation parameters were the setting of the band weights in the gradient and region growing. Five different band weights were set up for different situations and are listed in Table 1.

#### Setting merging parameters

In Table 2, the band weights setting corresponds to the band weights in Table 1 for the merging parameters. The mean difference and standard deviation difference parameters were set up according to three bands mean and standard (density, height, and aerial photo classification) extracted from DHH. The merging operation should be performed at least four times to reduce the small segmentation area. In addition, the setting of the merging value increased accordingly. In general, the merging parameters setting was the same for the different stand automatic segmentation because the mean and standard deviation of three bands was the same every time. The merging parameters setting was consistent for all seven experiments sets. The maximum area and small segment area settings were the same because the study area was fixed.

#### Segmentation results

**Figure 4:**
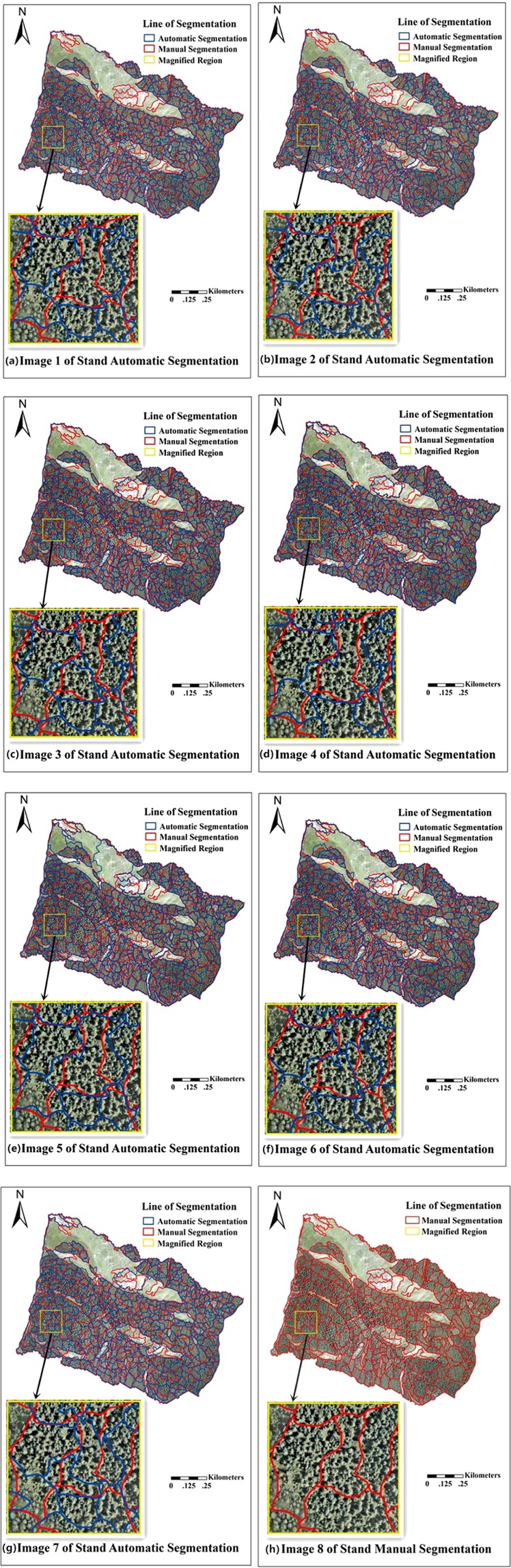
Results of stand automatic segmentation under different parameters.

The stand automatic segmentation operation was completed using ArboLiDAR software (http://www.arbonaut.com/en/). The smoothing and merging operations of small corresponding patches can be performed to make the results more fluent and easy to observe after standing automatic segmentation. The results of seven segmentation processes based on different parameters are shown in Fig. 4. In order to better present the automatic segmentation results, we added a line for manual segmentation as a contrast. Manual segmentation was based on the actual field situation, the forest map provided by the local forestry department, the aerial image, and the measured parameters of the study area in 2008 and 2010. Therefore, the manual segmentation result reliability was high. These results can serve as a reference to evaluate the automatic segmentation results.

Table 3 is a comparison of the segmentations stand number conducted after manual and automatic segmentation. It can be seen that the number of manual is less than that of automatic segmentation. The number of segmentations is almost the same in the seven groups.

**Table 3:** Results of stand segmentation number.

Mark Number	Segmentation Stand Number
Manual Segmentation	332
Automatic Segmentation 1	376
Automatic Segmentation 2	378
Automatic Segmentation 3	380
Automatic Segmentation 4	383
Automatic Segmentation 5	381
Automatic Segmentation 6	380
Automatic Segmentation 7	386

Figure 4 presents the results of the seven automatic and manual segmentations. The stand automatic segmentation results are shown in blue, and the manual segmentation line is shown in red. Compared with the different results of the automatic segmentation, the results showed that the automatic segmentation of image 5 was the best, followed by image 1 and image 7. The automatic segmentation results are not significant in images 2–6. In the magnified region of the selected part in every segmentation image, the automatic segmentation result was not the same. However, compared with the manual segmentation result the automatic segmentation results were more detailed and had more standpoints. After each automatic segmentation, it was found that the forest stand number was approximately 380, and the manual segmentations number was 332. The stand segmentations number also showed that the automatic segmentation was finer and more comprehensive. However, through a comparison between the results of each automatic segmentation, it was found that the segmentation features in details were still significantly different from each other. The automatic segmentation result in image 5 was obviously better than the other automatic segmentation results. It was noticeable that the segmentation effect of the non-forest land and low vegetation was apparent, and there was no uniform division of non-forest into a stand. At the same time, the automatic segmentation result was close to the manual segmentation result. Image 1 and image 7 showed no significant difference with image 5 in terms of the forest segmentation. However, the results in the segmentation of non-forest land and low vegetation were not very good. Image 2, 3, 4, and 6 had a good effect on the segmentation of a forest land small area, but it was not very satisfactory in the overall segmentation result, and the results were very different from the manual segmentation result. In addition, those four kinds of automatic segmentations were not very detailed in the non-forest land and low vegetation area.

### Results of stand parameters extracting

**Figure 5:**
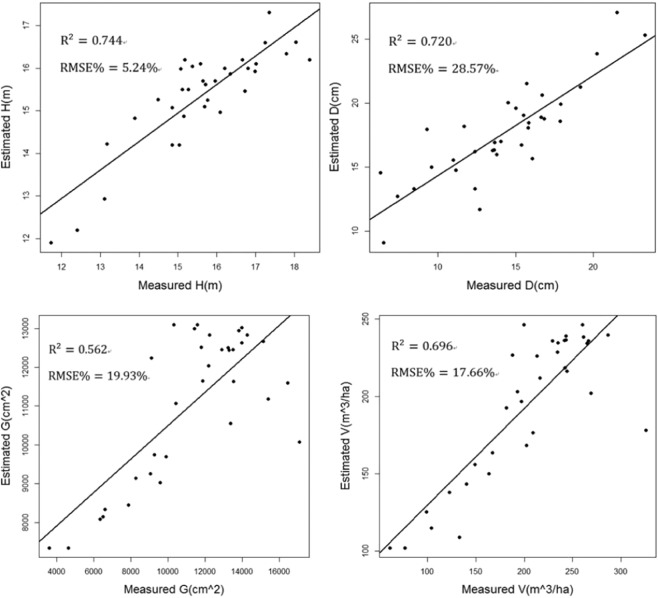
Scatter plots for forest parameters.

**Figure 6:**
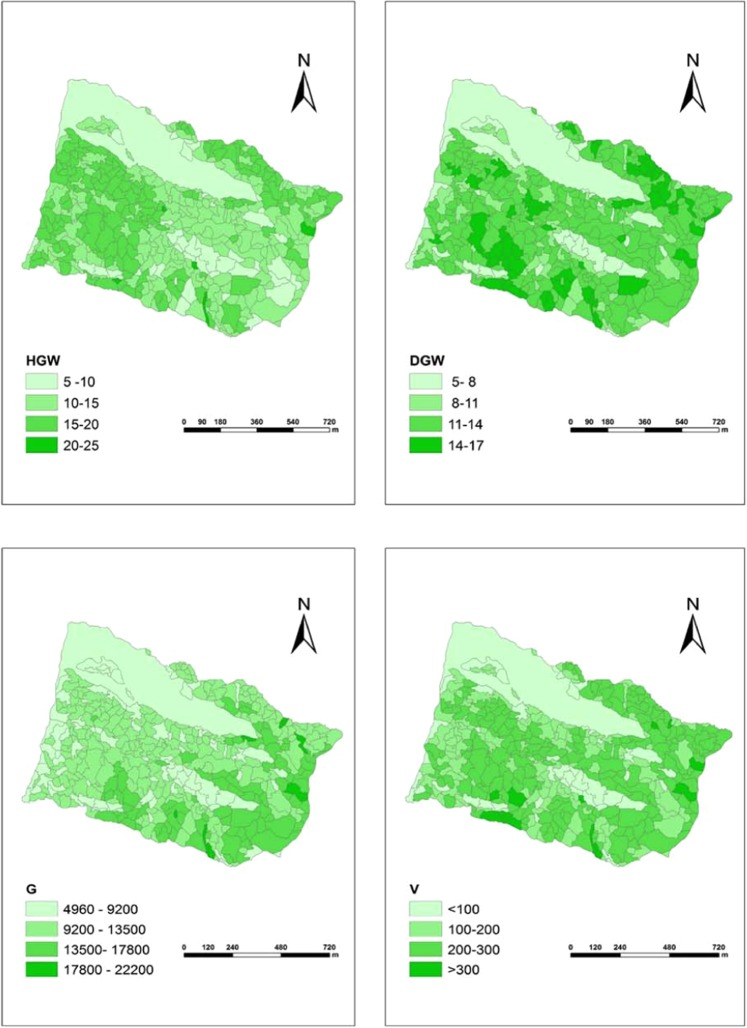
Spatial distribution of the stand parameters. These maps can also serve as a reference for forest management.

Table 4 presents the accuracy of the results. Figure 5 shows scatter plots of the plot-level field-based measured versus LiDAR-based retrieved parameters with linear fits. Figure 6 shows the maps of four stands parameters distribution in the study area. For the forest parameter H, the measured value derived from the plot data and the estimated value obtained from the Airborne LiDAR data have a strong correlation, with R^2^ measure of variation being 0.744, with a relative RMSE equal to 5.24%, with a relative Bias about 1.64%. For the parameter D, the R^2^ correlation measure is 0.720, relative Bias is 24.27%, and relative RMSE is 28.57%. For the parameter G, the R^2^ is 0.562, and Bias equal to 1.89%, RMSE equal to 19.93%. The Volume parameter (V) also have a correlation with R^2^ 0.696, relative RMSE equal to 17.66%, relative Bias equal to 4.2%. For all of the parameters, the P-value significance measurement is less than 0.001. The relative RMSE and relative Bias value are smaller, supporting a high confidence level in the results. An interesting observation arising from Fig. 4 is that, in this study area, most of the region has an H value that ranges between 15 and 25 meters. D value, meanwhile, ranges between12 and 17centimeters. The value of V ranges between 200 and 300 m^3^/ha. The reason for this situation is that the tree species belong to mature forest in this area, so the height and DBH of trees are large. At the same time, the tree species are dense and there are more trees in the fixed sample plot, the volume is large.

**Table 4:** Precision information of stand parameters.

Parameters	Units	R^2^	Adjusted R^2^	P-value	RMSE%	Bias%
H	m	0.744	0.736	0.000	5.24%	1.64%
D	cm	0.720	0.710	0.000	28.57%	24.27%
G	m^2^	0.562	0.549	0.000	19.93%	1.89%
V	m^3^/ha	0.696	0.687	0.000	17.66%	4.20%

## Discussion

This study attempted to evaluate the ArboLiDAR application in the north-west of China. We used an automatic stand segmentation technique based on a gradient algorithm and a region growing algorithm. In the algorithm, the segmentation parameters setting is the key factor that determines the segmentation results.

From the stand segmentation results, the different segmentation parameters setting makes the stand automatic segmentation effect very different. In general, the combination with the automatic stand segmentation settings parameters showed that the proportion of density, height, and aerial photo classification were the same in the stand automatic segmentation for the study area. It needs to consider these three factors and cannot highlight the privileges of only one particular factor. The stand automatic segmentation is a comprehensive consideration of all forest characteristics aspects and the same as manual segmentation. The automatic segmentation result in image 5 is closer to the manual segmentation result. In addition, image 1 and image 7 can be used to estimate the forest parameters in forest inventory if only the forest area segmentation result is considered, which means, without considering the non-forest area result.

From the stand parameters extracting results, forest parameters H, D, V obtained from the model have a strong correlation with their corresponding field parameters and obtained an R^2^ correlation measure more than 0.6. These indicated that the Sparse Bayesian regression is suitable for generating useful information about the kind of area in this study. Overall these results not only indicate that the Airborne LiDAR data can retrieve forest stand parameters with a high degree of precision but also demonstrate the ArboLiDAR effectiveness to process and obtain forest structure information of this study area. This study also generated the maps of each forest parameters distribution. This being the case, targeted forest-tending, one of the most important forest management activities conducted by local wardens and managers^47^, has the potential to be significantly assisted through reference to these maps. This kind of information is also useful to various forestry departments when trying to determine which method should be adopted for forest management. Some of the information, for instance, the volume, is also of potential interest for the assessment of such things as biomass, the carbon cycle and the atmosphere^48^.

Airborne LiDAR data have been applied to forest inventory widely, but which model can achieve good performance in practice still explored by researchers. This paper investigated a way of using the software ArboLiDAR to run a stand delineation algorithm that is able to combine Airborne LiDAR data with aerial photography. This function was suitable to be promoted in China, especially conducting the forest inventory at large range area, which can replace the manual method to delineate stands boundary. Besides, a way of using Sparse Bayesian regression to estimate forest stand parameters was also presented. Estimating forest parameter such as H, D, G, and Stand volume (V) achieved good precision. For the same study area, another research team conducted a similar forest inventory research, HeQisheng *et al*.^48^, obtained forest parameters using stepwise multiple regression models, results shown that height, stand density and crown width, with R^2^ precision measurement for H of 0.729, and for Diameter at Breast Height (D) of 0.588. It can be seen that the results obtained in this study are at least as good as these, if not better.

There are some deficiencies in the study. First, the point cloud data and aerial data classification accuracy will affect the stand automatic segmentation results in data preprocessing. Second, the segmentation parameters set can only be applied to the specific region. This means that appropriate parameter selection will be required for different research areas. Finally, although the Dayekou forest is a typical forest region in Northwest China, it can only represent parts of China and not all forests in the country. Based on the results and shortcomings of this research, we will focus on the above problems, avoid errors in the study, perform a deeper analysis of the software principle, and make our approach more adaptable so that it can be applied to additional Chinese forest areas.

## Conclusion

Using Airborne LiDAR data, field data from the DayeKou forest zone, and aerial photo used as a forest mask, the GARGA algorithm has been used to segment stands borders, the Sparse Bayesian regression approach has been used to produce various forest parameters estimates over the study area. Forest parameters such as H, D, G, and V have been estimated and achieved high precision. Therefore, conclusions obtained from the results are: 1) A GARGA algorithm is able to obtain good segmentation results using both Airborne LiDAR data and aerial photography when applied to stand segmentation; 2) Airborne LiDAR data is able to retrieve forest stand characteristics with high precision when a Sparse Bayesian regression methodology is adopted; and 3) The LiDAR software, ArboLiDAR, which was developed for forest inventory, has a potential to be used in the northwest of China, because it delineates stands automatically with high accuracy levels. This powerful functionality would seem to offer clear benefits to the Chinese community. When it comes to estimating forest parameters the software also performs well but has some limitations because researchers are not currently able to gain access to the source code or the data structure. In the future, our studies will concentrate on exploring how to develop even more advanced methods for the remote sensing of China forest characteristics.
